# Is environmental enrichment effective in modulating autophagy markers in the brain exposed to adverse conditions? A systematic review

**DOI:** 10.3389/fncel.2025.1624500

**Published:** 2025-06-25

**Authors:** Clarice Beatriz Gonçalves Silva, Matheus Santos de Sousa Fernandes, Debora Dantas Nucci Cerqueira, Gabriela Carvalho Jurema Santos, Fatma Hilal Yagin, Yalin Aygun, Georgian Badicu, Fabiana S. Evangelista, Pablo Prieto-González, Fabrício Oliveira Souto, Ashit Kumar Dutta, Sameer Badri Al-Mhanna, Abedelmalek Kalefh Tabnjh

**Affiliations:** ^1^Keizo Asami Institute, Federal University of Pernambuco, Recife, Brazil; ^2^Postgraduate Program in Biology Applied to Health, Federal University of Pernambuco, Recife, Brazil; ^3^Postgraduate Program in Nutrition, Physical Activity and Phenotypic Plasticity—UFPE/CAV, Vitória de Santo Antão, Brazil; ^4^Department of Biostatistics, Faculty of Medicine, Malatya Turgut Ozal University, Malatya, Türkiye; ^5^Department of Sport Management, Faculty of Sport Sciences, Inonu University, Malatya, Türkiye; ^6^Department of Physical Education and Special Motricity, Transilvania University of Brasov, Brasov, Romania; ^7^School of Arts, Science and Humanities, University of São Paulo, São Paulo, Brazil; ^8^Sport Sciences and Diagnostics Research Group, GSD-HPE Department, Prince Sultan University, Riyadh, Saudi Arabia; ^9^Department of Computer Science and Information Systems, College of Applied Sciences, AlMaarefa University, Riyadh, Saudi Arabia; ^10^Center for Global Health Research, Saveetha Medical College and Hospitals, Saveetha Institute of Medical and Technical Sciences, Saveetha University, Chennai, India; ^11^Department of Physiology, School of Medical Sciences, Universiti Sains Malaysia, Kubang Kerian, Malaysia; ^12^Department of Higher Studies, Al-Qasim Green University, Babylon, Iraq; ^13^Department of Cariology, Institute of Odontology, Sahlgrenska Academy, University of Gothenburg, Gothenburg, Sweden; ^14^Department of Applied Dental Sciences, Faculty of Applied Medical Sciences, Jordan University of Science and Technology, Irbid, Jordan

**Keywords:** enriched environment, cellular autophagy, central nervous system, metabolism, immune response

## Abstract

Autophagy is a key regulator of cellular homeostasis and neuronal survival, particularly under adverse physiological conditions. Environmental enrichment (EE), a non-pharmacological intervention providing enhanced sensory, cognitive, and motor stimulation, may modulate autophagic processes in the brain. This systematic review aimed to synthesize preclinical findings on the effects of EE on autophagy markers in rodent models subjected to diverse adverse conditions. A literature search across PubMed, Scopus, ScienceDirect, and embase yielded eight eligible studies meeting inclusion criteria. EE was found to be generally associated with upregulation of key autophagic markers such as Beclin-1, LC3-II/LC3-I ratio, cathepsins, p62, p-TFEB, and LAMP-1 across brain regions including the cortex, hippocampus, and penumbral area. However, reductions in some markers were also observed, indicating that the modulatory effects of EE are context-dependent and may vary with disease model, brain region, or EE protocol duration. These findings suggest that EE holds promise as an adjunctive strategy to modulate autophagy and mitigate neurodegeneration, though heterogeneity in study design and outcomes warrants caution during interpretation. Further mechanistic and sex-specific studies are needed to clarify the therapeutic relevance of EE-induced autophagic modulation.

## 1 Introduction

Autophagy is a fundamental catabolic process required for the maintenance of cellular homeostasis and the regulation of diverse physiological functions. It involves degrading and recycling cytoplasmic components, including organelles, proteins, and cellular debris (Liu et al., 2023). In the central nervous system, autophagy plays a critical role in tissue recovery following pathological insults such as stroke, infections, cellular damage, and pathogen invasion (Wolf et al., 2019). The autophagic process involves a tightly regulated sequence of events, including autophagosome formation, fusion with lysosomes, and subsequent maturation into the autolysosomal complex (Liu et al., 2023).

Given its essential role in cell survival, identifying stimuli that effectively modulates autophagic activity is of considerable scientific interest. In recent decades, the regulation of autophagic flux has received increasing attention as a potential contributor influencing outcomes in various pathological conditions, including cerebral ischemia (Sun et al., 2018). However, the role of modulating autophagic markers on these outcomes remains controversial, as the effects appear to vary depending on the specific brain region and the underlying pathophysiological context. For instance, studies have reported neurological recovery following inhibition of autophagy via the PI3K/AKT/mTOR signaling pathway (Li et al., 2023). Conversely, Yang et al. (2022) reported that inducing autophagy through mTOR pathway inhibition enhances the integrity of the blood-brain barrier (BBB) (Yang et al., 2022). These contrasting findings highlight significant gaps in our understanding of how autophagy is regulated in response to neurological insults and emphasize the context-dependent nature of its role in disease progression and recovery.

In this context, environmental factors play a key role in modulating responses to adversity at molecular, cellular, and systemic levels (Yang et al., 2025; Singhal et al., 2014; MacGillivray and Kollmann, 2014). EE has emerged as a promising non-pharmacological intervention capable of enhancing autophagic responses. EE involves exposing animals to enhanced sensory, cognitive, and social stimuli through larger, stimulus-rich environments and novel objects (Sun et al., 2018). The beneficial effects of EE on cognitive and behavioral parameters are well established, with studies demonstrating its capacity to reduce neuronal apoptosis (Li et al., 2023), and to promote neurogenesis and astrocyte proliferation (Yang et al., 2022). The modulatory effects of EE on neurophysiological processes are mediated by increased exploratory behavior, which subsequently enhances voluntary physical activity (de Sousa Fernandes et al., 2022; Fernandes et al., 2023). This heightened activity upregulates signaling pathways involved in the expression of neurotrophic factors, including brain-derived neurotrophic factor (BDNF) and its receptor, tropomyosin receptor kinase B (TrkB), contributing to neuroplasticity (Ismail et al., 2024). Furthermore, the positive regulation promoted by EE in mechanisms associated with neuroplasticity (neurogenesis and synaptogenesis) can protect the brain against inflammatory damage (de Sousa Fernandes et al., 2022; Bailey et al., 2022).

EE may influence neuronal autophagic activity by modulating the expression of autophagy-related genes, such as Beclin-1, and by altering the LC3-I/LC3-II ratio—an established marker of autophagosome formation (Yang et al., 2025). Experimental evidence supports the capacity of EE to regulate autophagic flux across different adverse contexts. For example, Deng et al. (2021) reported increased autophagic activity in the brains of animals exposed to EE following stroke. Similarly, Zhang et al. (2024) reported that EE conferred neuroprotection in sleep-deprived male C57BL/6J mice by inhibiting excessive autophagy in the forebrain. These findings highlight the relevance of EE in modulating brain autophagy and experimental models of neurological insult.

Despite the growing interest in the role of environmental enrichment (EE) in regulating autophagy, its effects on autophagic markers across different pathological contexts have not yet been systematically synthesized. This review aims to fill this gap by evaluating current preclinical evidence on EE-induced modulation of autophagic processes, with a focus on the underlying mechanisms in the brain under adverse physiological conditions.

## 2 Methods

This review is synthesized in accordance with the Preferred Reporting Items for Systematic Reviews and Meta-Analyses (PRISMA) (Page et al., 2021). This systematic review was not registered.

### 2.1 Eligibility criteria

Eligible studies were those that: (1) used different rodent species; (2) evaluated outcomes in various brain areas; (3) assessed autophagy markers; (4) employed environmental enrichment as an intervention strategy; (5) used physical exercise as part of the overall EE protocol, and not alone; (6) investigated different conditions of adverse environmental exposure, such as drugs, diseases, and clinical conditions; (7) included a control group; (8) were published in English. Review articles, letters to the editor, unpublished studies, and abstracts were excluded. Additionally, studies that did not report the outcomes of interest were considered ineligible. No time limitations for publication or minimum follow-up duration in the intervention were applied.

### 2.2 Information sources and search strategy

The article search was conducted in January/2025 across the PubMed/MEDLINE, Scopus, ScienceDirect, and Embase databases. CBGS; MSSF and GCJS independently conducted the literature search, extraction, and validation stages. The following search strategy was used: [(“Environmental Enrichment”) OR (“Enriched Environment”)] AND [((“Autophagy”) OR (“Autophagy, Cellular”)) OR (“Cellular Autophagy”)]. Necessary adaptations were made to the databases. No filter was applied at this stage.

### 2.3 Selection and data collection process

The selection and data collection process were conducted by two independent authors (CS and MS), with disagreements resolved by a third author (FSo). This step was performed using Endnote X20 software (Clarivate Analytics, Philadelphia, United States). Duplicates were removed. Initially, the title and abstract of each article were reviewed, followed by a full-text evaluation of the selected articles.

### 2.4 Data items

The following information was extracted from each study: (1) general information—author, year; (2) sample characteristics—species, age, sex, *n* per cage; (3) environmental enrichment protocol—inanimate objects, dimensions (length, width, height), exposure time; (4) outcomes—adverse condition, brain area, biological material, analysis technique; (6) main findings—autophagy outcomes.

### 2.5 Methodological quality assessment

The methodological quality of the studies was assessed using the SYRCLE strategy (Hooijmans et al., 2014). The tool consists of 10 questions that assess methodological criteria, such as animals’ allocation sequence, adjustment for confounding factors, sample randomization, blinding, presence of incomplete results, and problems resulting in risk of bias. The questions were answered with the options of “Yes,” “No” or “Unclear.” When the answer was “Yes,” a score was given; when the answer was “No” or “Unclear,” no score was given. Overall scores for each article were calculated on a scale of 0 to 10 points, with the quality of each study being classified as high (8 to 10), moderate (5 to 7) or low (<5).

## 3 Results

**Figure 1:**
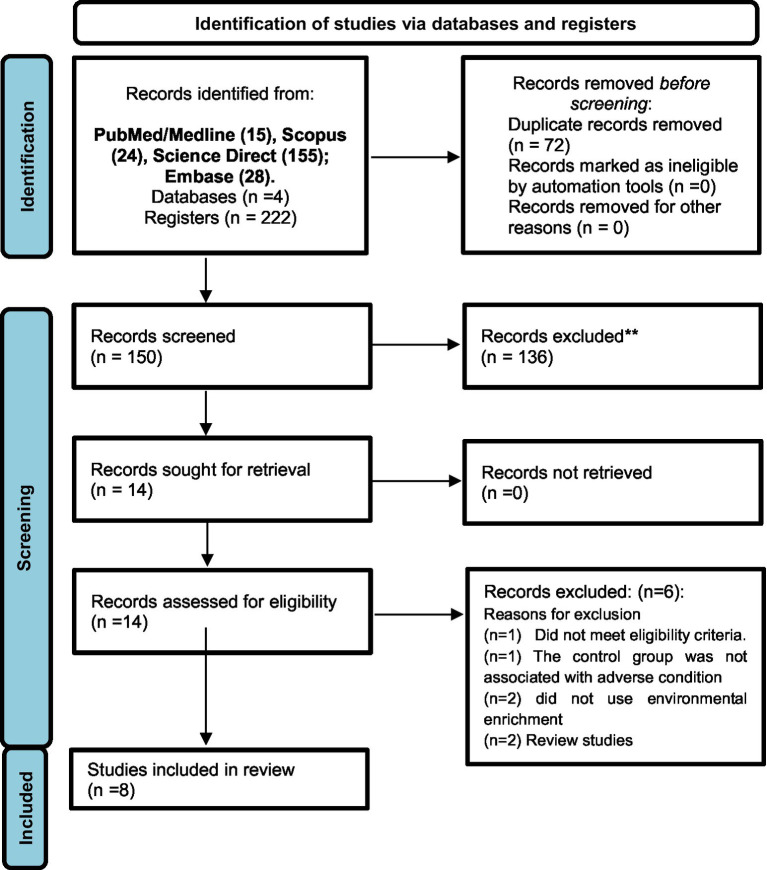
PRISMA flow diagram of each database or registry searched to obtain the studies included in this systematic review. For more information, visit: http://www.prisma-statement.org/.

A total of 222 articles were initially identified across the following databases: PubMed (*n* = 15); Scopus (*n* = 24); Science Direct (*n* = 155); Embase (*n* = 28). Following the screening process, only eight studies met the eligibility criteria and were included in the final analysis (Figure 1). The assessment of methodological quality revealed that all included studies demonstrated moderate methodological rigor, with an average quality score of 6 points. All studies employed an appropriately generated and applied allocation sequence, which was both concealed and randomized, and the selection of animals for outcome assessment was conducted randomly. The study reports did not exhibit selective outcome reporting and did not appear to present other issues that could result in a high risk of bias. However, none of the studies reported that the researchers were blinded to the interventions each animal received during the experiment. Additionally, it was unclear whether the groups were comparable at baseline and how incomplete data were addressed (Table 1).

**Table 1:** Methodological quality assessment.

Author, year	Q1	Q2	Q3	Q4	Q5	Q6	Q7	Q8	Q9	Q10	Score
Deng et al. (2021)	Y	U	Y	Y	N	Y	N	U	Y	Y	6
Han et al. (2023)	Y	U	Y	Y	N	Y	N	U	Y	Y	6
Zhang et al. (2022)	Y	U	Y	Y	N	Y	N	U	Y	Y	6
Takahashi et al. (2014)	Y	U	Y	Y	N	Y	N	U	Y	Y	6
Xu et al. (2020)	Y	U	Y	Y	N	Y	N	U	Y	Y	6
Xu et al. (2022)	Y	U	Y	Y	N	Y	N	U	Y	Y	6
Xu et al. (2023)	Y	U	Y	Y	N	Y	N	U	Y	Y	6
Zhang et al. (2024)	Y	U	Y	Y	N	Y	N	U	Y	Y	6

Q1: Was the allocation sequence adequately generated and applied? Q2: Were the groups similar at baseline or were they adjusted for confounders in the analysis? Q3: Was the allocation to the different groups adequately concealed during? Q4: Were the animals randomly housed during the experiment? Q5: Were the caregivers and/or investigators blinded from knowledge which intervention each animal received during the experiment? Q6: Were animals selected at random for outcome assessment? Q7: Was the outcome assessor blinded? Q8: Were incomplete outcome data adequately addressed? Q9: Are reports of the study free of selective outcome reporting? Q10: Was the study apparently free of other problems that could result in high risk of bias? Y, yes; N, no; U, unclear.

Details regarding sample characteristics and environmental enrichment protocols used across studies are summarized in Table 2. The selected studies were published between 2014 and 2024. Different species of animals were used: Sprague Dawley (*n* = 5) (Xu et al., 2020; Xu et al., 2022; Xu et al., 2023; Deng et al., 2021; Han et al., 2023), C57BL/6J (*n* = 2) (Zhang et al., 2022; Zhang et al., 2024), Wistar (*n* = 1) (Takahashi et al., 2014), with ages ranging from 4 weeks (Han et al., 2023) to 12 weeks (Zhang et al., 2022). All animals used were male. The number of animals per cage was between 5 (Zhang et al., 2024) and 12 (Xu et al., 2020; Xu et al., 2022; Xu et al., 2023) per box. Different inanimate objects were used to compose the EE protocol. Most studies used running wheels (*n* = 6) (Xu et al., 2020; Xu et al., 2022; Xu et al., 2023; Han et al., 2023; Zhang et al., 2024; Takahashi et al., 2014), followed by tunnels (*n* = 5) (Xu et al., 2020; Xu et al., 2023; Deng et al., 2021; Zhang et al., 2024; Takahashi et al., 2014), toys (*n* = 5) (Xu et al., 2020; Xu et al., 2022; Deng et al., 2021; Han et al., 2023; Takahashi et al., 2014), platforms (*n* = 4) (Xu et al., 2020; Xu et al., 2023; Deng et al., 2021; Han et al., 2023), seesaw (*n* = 3) (Xu et al., 2023; Han et al., 2023; Zhang et al., 2024), balls (*n* = 3) (Xu et al., 2023; Han et al., 2023; Takahashi et al., 2014) and blocks (*n* = 3) (Deng et al., 2021; Zhang et al., 2022; Takahashi et al., 2014). Swing (*n* = 2) (Han et al., 2023; Zhang et al., 2024) and ladder (*n* = 2) (Deng et al., 2021; Zhang et al., 2024) were also used. Chains (Deng et al., 2021), pipes, mazes, shelters, nesting (Han et al., 2023), houses, channels, fitness rings (Zhang et al., 2024), chews, huts, retreats, holes (Takahashi et al., 2014), and sports room were used in only one study (Zhang et al., 2022). The housing dimensions ranged from 40 × 30 × 40 cm (Zhang et al., 2024) to 95 × 75 × 45 cm (Xu et al., 2020; Xu et al., 2022; Xu et al., 2023). The exposure time to the enriched environment protocol ranged from 7 days (Zhang et al., 2022) to 6 weeks (Xu et al., 2020).

**Table 2:** Sample characteristics and environmental enrichment protocol.

Author, year	Species, age	Sex	*n* per cage	Environmental enrichment protocol and housing dimensions		Exposure time
				Inanimate objects	(Length, width, height)	
Deng et al. (2021)	Sprague Dawley rats, 9–10 wks old	M	10–12	Toys, ladders, plastic tunnels, colored blocks, tubes, platforms, and chains	85 × 50 × 50 cm	5 wks
Han et al. (2023)	Sprague Dawley rats, 4 wks old	M	10	Running wheel, plastic toys, hanging swing, seesaw, wooden jumping platforms, transparent pipes, shuttle mazes, shelters, colored rattan balls, and nesting material	40 × 30 × 60 cm	4 wks
Zhang et al. (2022)	C57BL/6J mice, 8 wks old	M	5	Running wheels, inclined ladders, L-shaped tunnels, crawling tubes, seesaws, small houses, rainbow channels, swings, and fitness rings.	40 × 30 × 40 cm	2 wks
Takahashi et al. (2014)	Wistar rats, 6 wks old	M	6	Running wheels, bone-shaped toys, paper and plastic tunnel, wooden blocks, plastic, cyclone chews, hut, retreat, plastic crow-ball, and holes.	40 × 54 × 30 cm	2 wks
Xu et al. (2020)	Sprague Dawley rats, adult	M	12	Running wheels, toys, tunnels, platforms made of metal or wood	95 × 75 × 45 cm	6 wks
Xu et al. (2022)	Sprague Dawley rats, NI	M	12	Running wheels, differently shaped toys, plastic tubes and plastic balls	95 × 75 × 45 cm	3 wks
Xu et al. (2023)	Sprague Dawley rats, 6 wks old	M	6	Tunnels, plastic or wood, seesaw, PVC platforms, and small plastic balls	95 × 75 × 45 cm	4 wks
Zhang et al. (2024)	C57BL/6J mice, 8–12 wks old	M	11	Running wheel, colored blocks, run room, warped tube, and sports room	80 × 60 × 40 cm	7 days

cm, centimeters; NI, not informed; wks, weeks; M, male; F, female.

Adverse conditions, brain area, biological material, analytical techniques, and autophagy-related outcomes are detailed in Table 3. The animals were exposed to different conditions, such as stroke (*n* = 3) (Deng et al., 2021; Han et al., 2023; Zhang et al., 2022), sleep deprivation (*n* = 1) (Zhang et al., 2024), inescapable stress (*n* = 1) (Takahashi et al., 2014), vascular occlusion (*n* = 1) (Xu et al., 2020), chronic unpredictable mild stress (CUMS) (*n* = 1) (Xu et al., 2022) and chronic cerebral hypoperfusion (*n* = 1) (Xu et al., 2023). Different brain areas were evaluated, with the hippocampus being the most explored area (*n* = 4) (Xu et al., 2020; Xu et al., 2022; Xu et al., 2023; Takahashi et al., 2014), followed by the cortex (*n* = 2) (Han et al., 2023; Zhang et al., 2022), penumbral area (*n* = 1) (Deng et al., 2021) and forebrain (*n* = 1) (Zhang et al., 2024). All studies used protein evaluation as biological material by western blotting (*n* = 7) (Xu et al., 2020; Xu et al., 2022; Xu et al., 2023; Deng et al., 2021; Han et al., 2023; Zhang et al., 2022; Zhang et al., 2024), immunoblotting (*n* = 1) (Takahashi et al., 2014) or ELISA (*n* = 1) (Xu et al., 2022). Different autophagy markers were investigated, such as LC3-II/LC3-I (*n* = 6) (Xu et al., 2020; Xu et al., 2022; Xu et al., 2023; Deng et al., 2021; Han et al., 2023; Zhang et al., 2024), p62 (*n* = 5) (Xu et al., 2022; Xu et al., 2023; Han et al., 2023; Zhang et al., 2022; Zhang et al., 2024), Beclin-1 (*n* = 4) (Xu et al., 2020; Xu et al., 2022; Deng et al., 2021; Han et al., 2023), LAMP-1 (*n* = 3) (Xu et al., 2020; Xu et al., 2023; Deng et al., 2021), LC3-II (*n* = 2) (Zhang et al., 2022; Takahashi et al., 2014), cathepsin-B, cathepsin-D, soluble SQSTM1, insoluble SQSTM1, ubiquitin, p-TFEB, TFEB (*n* = 1) (Deng et al., 2021), and p-mTOR/mTOR (*n* = 1) (Zhang et al., 2024).

**Table 3:** Impacts of environmental enrichment on oxidative stress and antioxidant outcomes in experimental models subjected to normal environmental conditions.

Author, year	Adverse condition	Brain area	Biological material	Analysis technique	Autophagy markers
Deng et al. (2021)	Stroke	Penumbral area	Protein	Western blotting	↑ Beclin-1; ↑ LC3-II/LC3-I; ↑ LAMP-1; ↑ Cathepsin-B; ↑ Cathepsin-D ↓ Soluble SQSTM1; ↓ Insoluble SQSTM1; ↓ Ubiquitin
Han et al. (2023)	Stroke	Cortex	Protein	Western blotting	↓ LC3-II/LC3-I; ↓ Beclin-1; ↓ LC3; ↑ p62
Zhang et al. (2022)	Sleep deprivation	Forebrain	Protein	Western blotting	↑ p62; ↑ p-TFEB; ↓ TFEB; ↓ LC3-II/LC3
Takahashi et al. (2014)	Inescapable stress	Hippocampus	Protein	Immunoblotting	↓ LC3-II
Xu et al. (2020)	Vascular oclusion	Hippocampus	Protein	Western blotting	↑ LAMP-1; ↑ Beclin-1; ↑ LC3-II/LC3-I
Xu et al. (2022)	CUMS	Hippocampus	Protein	Western blotting/elisa	↑ Beclin-1; ↑ LC3-II/LC3-I; ↓ p62
Xu et al. (2023)	Chronic cerebral hypoperfusion	Hippocampus	Protein	Western blotting	↑ LAMP-1; ↑ LC3-II/LC3-I; ↓ p62
Zhang et al. (2024)	Stroke	Cortex	Protein	Western blotting	↓ p62; ↓ p-mTOR/mTOR; ↑ LC3-II

CUMS, chronic inescapable stress; LC, light-chain protein; LAMP-1, lysosomal-associated membrane protein 1; p-mTOR, phosphorylated mammalian target of rapamycin; mTOR, mammalian target of rapamycin; SQSTM1/p62, sequestosome 1; TFEB, transcription factor EB; p-TFEB, phosphorylated TFEB; ↔: no significant difference (*p* > 0.05); increase; ↓: significant decrease (*p* < 0.05).

Autophagy-related outcomes indicate that environmental enrichment led to an increase in Beclin-1 expression in the penumbral region and hippocampus (Ismail et al., 2024; Bailey et al., 2022; Hooijmans et al., 2014). In contrast, Han et al. (2023) reported a reduction in Beclin-1 levels in the cortex. The expression of LC3 and LC3-II proteins was found to decrease following EE in the cortex (Xu et al., 2020) and hippocampus (Deng et al., 2021) in models of stroke and inescapable stress, respectively. Conversely, Zhang et al. (2022) observed an increase in LC3-II expression in the cortex of stroke-induced animals following EE. Regarding the LC3-II/LC3-I ratio, an increase was reported in the penumbral area and hippocampus in models of stroke (Hooijmans et al., 2014), vascular occlusion (Ismail et al., 2024), chronic unpredictable mild stress (Bailey et al., 2022), and chronic cerebral hypoperfusion (Page et al., 2021) after EE exposure. However, a reduction in this ratio was observed in the forebrain of sleep-deprived animals following EE (Xu et al., 2023).

EE was shown to increase the expression of LAMP-1 protein in animal models of chronic cerebral hypoperfusion (Page et al., 2021), vascular occlusion (Ismail et al., 2024), and stroke (Hooijmans et al., 2014) Additionally, EE led to a reduction in p62 protein levels under conditions of CUMS (Bailey et al., 2022), stroke (Xu et al., 2022), and chronic cerebral hypoperfusion (Page et al., 2021). In contrast, an increase in p62 was observed in the cortex following stroke (Xu et al., 2020).

Deng et al. (2021) investigated lysosomal and proteostasis-related proteins in the penumbral region under stroke conditions, reporting increased levels of cathepsin B and D, and decreased levels of both soluble and insoluble SQSTM1 (p62) and ubiquitin following EE exposure. Lastly, EE promoted an increase in phosphorylated TFEB (p-TFEB) and a concomitant reduction in total TFEB expression in the forebrain of sleep-deprived animals (Xu et al., 2023).

## 4 Discussion

**Figure 2:**
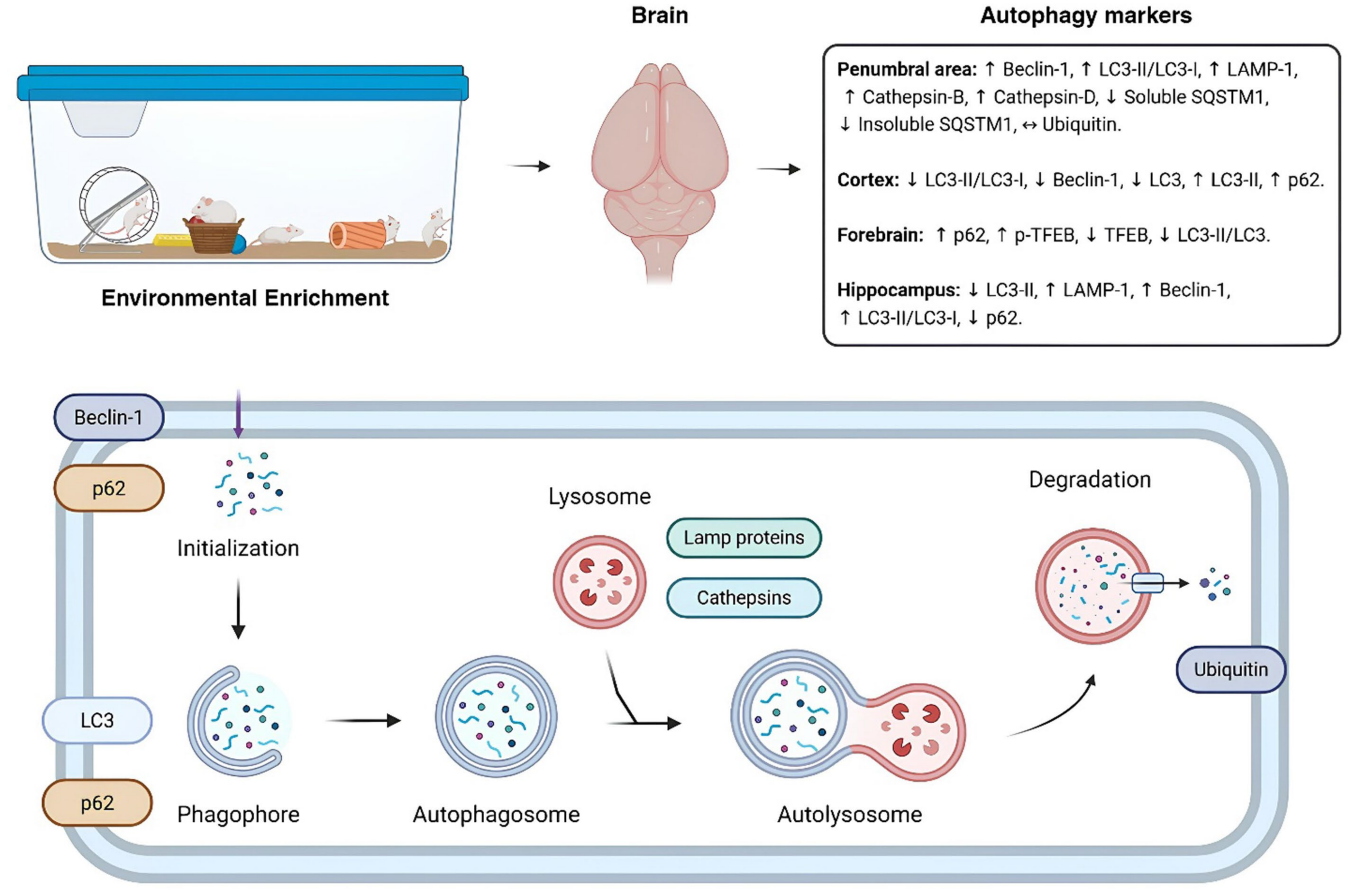
Impact of environmental enrichment on autophagy markers diagram of the autophagic flux within the cell. The figure shows the configuration of an environmental enrichment and its influences on autophagic markers in brain regions of the different models evaluated in the systematic review. Below, a summarized scheme of the markers evaluated within the autophagic flux. ↔: No significant difference (*p* > 0.05); ↓: Significant downregulation; (*p* < 0.05) ↑: Significant upregulation (*p* < 0.05). Autophagy markers description: LC, light-chain protein; LAMP-1, lysosomal-associated membrane protein 1; SQSTM1/p62, sequestosome 1; TFEB, transcription factor EB; p-TFEB, phosphorylated TFEB.

To our knowledge, this is the first systematic review to evaluate the effects of EE on the modulation of autophagic flux components in preclinical mouse models exposed to various adverse conditions. Our findings suggests that EE can modulate markers of autophagic flux, depending on the pathological context (see Figure 2).

EE has been found to change the levels of beclin-1 in the hippocampus, cortex, and penumbral area (Ismail et al., 2024; Bailey et al., 2022; Hooijmans et al., 2014; Xu et al., 2020). This is significant because Beclin-1 plays a crucial role in the formation, elongation, and maturation of autophagosomes, highlighting the importance of understanding the role of EE in initiating the autophagic process, as well as the functions of LC3-I and LC3-II (Li et al., 2021). The increased synthesis of Beclin-1 suggests the regulation of other molecules involved in this process, such as LAMP-1, p62, cathepsin B/D, ubiquitin, p-TFEB and TFEB, these molecules are not only related to autophagy, but also to the regulation of the immune system and the inflammatory response (Sanmarco et al., 2021; Xie et al., 2023).

Although this systematic review compiles evidence suggesting that EE influences autophagy under a variety of adverse conditions—promoting activation in regions such as the penumbra and cerebral cortex, while decreasing autophagy in areas such as the hippocampus and forebrain—the results are inconsistent and dependent on the region, adverse conditions studied, and duration of EE exposure. This variability highlights a fundamental uncertainty regarding the direct impact of EE on autophagy. The lack of coherent, region-specific mechanisms across studies questions the robustness of the observed effects and highlights the need for more targeted and methodologically sound research in this area.

EE is an effective rehabilitation intervention and can alter the progression of diseases in various neurodegenerative disease models, offering a dynamic approach to enhancing animal welfare and promoting behavioral enrichment (Xu et al., 2025). EE exposure can reduce the inflammatory response induced by cerebral ischemia-reperfusion injury, limit the extent of neuronal apoptosis, and ameliorate cognitive deficits (Fernandes et al., 2023; Xu et al., 2022). The molecular mechanisms by which EE influences neurodegenerative diseases are complex and involve several signaling pathways, including the activation of autophagy.

Transcription factor EB (TFEB) is considered the master transcriptional regulator of autophagy and lysosomal biogenesis (Settembre et al., 2012). TFEB is in the cytoplasm under basal cellular conditions and translocate to the nucleus in response to starvation, lysosomal stress, pathogen infections, ER stress, and exercise to promote organismal homeostasis (Franco-Juárez et al., 2022). Once activated, TFEB directly binds to the promoter sequences to augment the expression of autophagy-lysosome-related genes, promoting the biogenesis of lysosomes, autophagosomes, and their fusion with lysosomes to efficiently degrade complex molecules (Xu et al., 2023; Deng et al., 2021). Dysregulation of TFEB activity may contribute to the development of several diseases, including hepatic steatosis, neurodegenerative diseases, cancer, and inflammatory diseases (Deng et al., 2021).

Autophagy plays a key role in preventing stress as one of the major quality control mechanisms in the cell (Klionsky et al., 2021). The autophagic process can be subdivided into five phases: (1) initiation, (2) phagophore nucleation, (3) phagophore expansion and substrate selection, (4) autophagosome-lysosome fusion, and (5) lysosomal substrate degradation. All steps are regulated by autophagy-related genes (ATGs) (Klionsky et al., 2021; Eskelinen and Saftig, 2009).

During autophagy, certain proteins stand out, particularly microtubule-associated protein 1 light chain 3 beta (MAP1LC3B; also known as LC3), which is considered the primary marker of autophagic activity in cells (Galluzzi and Green, 2019). This is due to its essential role in the formation of autophagosomes, a hallmark of autophagy. In this process, the ATG12-ATG5: ATG16L1 complex, along with ATG4, ATG7, and ATG3, facilitates the conjugation of phosphatidylethanolamine (PE) to cytosolic LC3-I, generating the lipidated form LC3-II. LC3-II is subsequently incorporated into the autophagosome membrane, enabling substrate recognition through interaction with various autophagy receptors, including sequestosome 1 (SQSTM1/ p62) (Galluzzi and Green, 2019; Galluzzi et al., 2017).

Functional autophagic responses are crucial for preserving neuronal integrity following acute injury (Galluzzi et al., 2016). Exposure to EE has demonstrated various neuroprotective effects in many animal models (Sun et al., 2018), possibly associated with the regulation of autophagy. Deng et al. (2021) showed that rats submitted to middle cerebral artery occlusion (MCAO) and EE therapy enhanced Beclin-1 expression and LC3-II/LC3-I ratio, indicating the occurrence of autophagic process and minor neurological deficits. Xu et al. (2020) using an experimental model of chronic cerebral hypoperfusion (CCH), observed that the treatment with EE increased the expression of LAMP1, Beclin-1 and LC3 proteins, which could be associated with the improvement of autophagy dysfunction caused by CCH. Another study, using an experimental model of Parkinson’s disease, demonstrated that treatment with EE could enhance autophagy (Jang et al., 2018). The authors observed increased expression of LC3-II, Beclin-1, LAMP2, cathepsin L, and TFEB, alongside improvements in motor function in mice; however, this was accompanied by an increase in p62 levels (Jang et al., 2018).

However, evidence on the role of autophagy in EE-mediated protection are limited and conflicting, highlight the conflicting nature of the topic.

The onset of the “diseased state” associated with autophagy dysregulation may result from alterations in central aspects of multicellular organism biology (Han et al., 2023). Han et al. (2023), using an MCAO model, observed an increase in the LC3-II/LC3-I ratio and Beclin-1 expression, alongside a decrease in p62, indicating the activation and occurrence of the autophagic process. Furthermore, when MCAO animals were pretreated with EE, a decrease in LC3 and Beclin-1 expression and an increase in p62 were observed, suggesting enhanced autophagosome clearance (Xu et al., 2020).

This review highlights the dual role of EE in modulating autophagy, which appears to be influenced by the specific disease model, brain region analyzed, and duration of EE exposure. These findings underscore the complexity of EE-induced autophagic regulation and emphasize the need for further mechanistic studies to elucidate its molecular pathways. Despite the growing body of research investigating the effects of EE on autophagy under adverse conditions, the current literature remains fragmented and often contradictory. While some studies suggest that EE facilitates autophagy activation and contributes to disease modulation, others report reductions in autophagic marker expression, casting doubt on the consistency of these findings. This lack of consensus highlights a gap in the field.

Looking ahead, the integration of EE with existing therapeutic strategies may offer enhanced potential for the treatment of neurodegenerative diseases. EE may act synergistically with pharmacological agents, particularly those targeting the autophagic pathway, thereby improving treatment efficacy and promoting neuroprotection in clinical settings. Finally, we highlight that although EE advocates the use of voluntary physical activity or physical exercise, mainly through exploratory behavior as a component of its model, but not alone. Since physical exercise can be conceptualized as the performance of planned and structured physical activities with the main objective of improving physical fitness. Based on knowledge about these protocols, future studies can be planned and carried out, with the aim of comparing the different effects of these interventions on autophagy markers and their mechanisms in the brain and other tissues, with a lower rate of heterogeneity of the protocols. Thus, bringing better health conditions and quality of life.

## 5 Limitations and strengths

This study is the first systematic review to demonstrate how different EE protocols modulate autophagic flux in brain tissue across a range of pathological conditions. The findings indicate that the activation or inhibition of autophagy varies according to the specific experimental model used, opening new avenues for a deeper understanding of autophagic mechanisms at various stages of disease progression, in different brain regions, and under diverse protocols. In this context, the review highlights the potential of EE as an adjuvant therapy to conventional treatments, with the aim of enhancing patient recovery. It also underscores that EE pretreatment can improve neurological outcomes in experimental models of neurological disorders.

However, the evidence exploring the relationship between EE and autophagy—particularly in the context of prolonged exposure and protocol variations—remains limited. Additionally, the studies included in this review differ in terms of the experimental models employed, which makes it challenging to draw precise conclusions about the effects of the intervention. One of the major gaps in the literature is the predominant use of experimental models that include only male subjects, which hinders more accurate conclusions about the behavior of autophagic markers in females following EE. Furthermore, we encourage additional studies investigating the chronic impacts of enrichment on these markers to broaden the temporal perspective of EE. Furthermore, additional studies should investigate sex- and age-related differences to deepen our understanding of how autophagic flux mechanism’s function and are modulated under different physiological conditions, considering hormonal and senescence-related factors.

Despite these limitations, we highlight the importance of future research comparing a wider range of EE protocols and evaluating the intervention in combination with pharmacological treatments currently used in clinical practice, to better assess the potential of EE to enhance patient recovery. From this perspective, it is also necessary to examine the crosstalk between autophagic flux mechanisms and EE in relation to disease-associated behavioral outcomes, which were not the primary focus of this review.

## 6 Conclusion

In conclusion, the findings of this systematic review indicate that EE effectively modulates key markers of autophagic flux. Regulatory effects were observed on critical molecules involved in autophagosome formation, such as Beclin-1, LC3-I, and LC3-II, as well as on other intermediates linked to distinct stages of the autophagy pathway. These results suggest that EE has the potential to broadly influence autophagic processes and mitigate the detrimental effects of dysregulated or excessive autophagy, offering promising implications for therapeutic strategies and disease modulation.

This review advances our understanding of the mechanisms underlying EE-based interventions and their potential role in influencing disease progression and neural recovery. Moreover, the findings highlight the relevance of EE as an adjunctive, non-pharmacological strategy for enhancing clinical efficacy and improving patient outcomes. Nonetheless, further research is warranted to elucidate the precise mechanisms of EE across different disease models, brain regions, and intervention protocols.

## Data Availability

The original contributions presented in the study are included in the article/Supplementary material, further inquiries can be directed to the corresponding authors.
